# Acrolein, an endogenous aldehyde induces synaptic dysfunction in vitro and in vivo: Involvement of RhoA/ROCK2 pathway

**DOI:** 10.1111/acel.13587

**Published:** 2022-03-22

**Authors:** Zeyu Zhu, Junfeng Lu, Shuyi Wang, Weijia Peng, Yang Yang, Chen Chen, Xin Zhou, Xifei Yang, Wenjun Xin, Xinyi Chen, Jiakai Pi, Wei Yin, Lin Yao, Rongbiao Pi

**Affiliations:** ^1^ School of Medicine Sun Yat‐Sen University Guangzhou China; ^2^ School of Pharmaceutical Sciences Sun Yat‐Sen University Guangzhou China; ^3^ Department of Internal Medicine The Affiliated Tumor Hospital of Zhengzhou University Zhengzhou China; ^4^ Zhongshan School of Medicine Sun Yat‐Sen University Guangzhou China; ^5^ Key Laboratory of Modern Toxicology of Shenzhen Center for Disease Control and Prevention; ^6^ School of Pharmaceutical Sciences South China Research Center for Acupuncture and Moxibustion Guangzhou University of Chinese Medicine Guangzhou China; ^7^ Guangzhou Foreign Language School Guangzhou China; ^8^ Research Institute of Acupuncture and Moxibustion Shandong University of Traditional Chinese Medicine Jinan China; ^9^ International Joint Laboratory<SYSU‐PolyU HK> of Novel Anti‐Dementia Drugs of Guangzhou Guangzhou China; ^10^ Guangdong Province Key Laboratory of Brain Function and Disease Sun Yat‐sen University Guangzhou China

**Keywords:** acrolein, Alzheimer's disease, Fasudil, ROCK2, synaptic dysfunction

## Abstract

Acrolein, an unsaturated aldehyde, is increased in the brain of Alzheimer's disease (AD) patients and identified as a potential inducer of sporadic AD. Synaptic dysfunction, as a typical pathological change occurring in the early stage of AD, is most closely associated with the severity of dementia. However, there remains a lack of clarity on the mechanisms of acrolein inducing AD‐like pathology and synaptic impairment. In this study, acrolein‐treated primary cultured neurons and mice were applied to investigate the effects of acrolein on cognitive impairment and synaptic dysfunction and their signaling mechanisms. In vitro, ROCK inhibitors, Fasudil, and Y27632, could attenuate the axon ruptures and synaptic impairment caused by acrolein. Meanwhile, RNA‐seq distinct differentially expressed genes in acrolein models and initially linked activated RhoA/Rho‐kinase2 (ROCK2) to acrolein‐induced synaptic dysfunction, which could regulate neuronal cytoskeleton and neurite. The Morris water maze test and in vivo field excitatory postsynaptic potential (fEPSP) were performed to evaluate spatial memory and long‐term potential (LTP), respectively. Acrolein induced cognitive impairment and attenuated LTP. Furthermore, the protein level of Synapsin 1 and postsynaptic density 95 (PSD95) and dendritic spines density were also decreased in acrolein‐exposed mice. These changes were improved by ROCK2 inhibitor Fasudil or in ROCK2^+/−^ mice. Together, our findings suggest that RhoA/ROCK2 signaling pathway plays a critical role in acrolein‐induced synaptic damage and cognitive dysfunction, suggesting inhibition of ROCK2 should benefit to the early AD.

## Graphical abstract

Acrolein could induce fractures of dendritic spines and synaptic dysfunction both in vitro and in vivo. Synaptic dysfunction induced by acrolein is related to the RhoA/ROCK2 signaling pathway. Synaptic dysfunction induced by acrolein can be attenuated by knocking down ROCK2 or using ROCK inhibitor Fasudil.

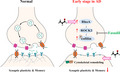

## 1 INTRODUCTION

Alzheimer's Disease (AD), the most common dementia, can be divided into familial AD (fAD) and sporadic AD (sAD), with the latter accounting for as high as about 95% ("2021 Alzheimer's disease facts and figures," 2021). Currently, there is still no effective treatment for AD, especially moderate and severe AD (Long & Holtzman, 2019). Meanwhile, a vast majority of those diagnosed patients are found in the middle and late stages. Therefore, early prevention, diagnosis, and treatment have been widely recognized as the effective solution to fight against AD (Mehta et al., 2017). Synaptic loss and dysfunction are the typical pathological changes that occur in the early stage of AD (Selkoe, 2002). Since synaptic dysfunction is closely linked to the severity of dementia, it is essential to study the etiology and intervention strategies of synaptic dysfunction (Torres et al., 2021).

Synapses play significant roles in the connection between neurons in neural networks and perform vitally important physiological functions (John & Reddy, 2021; Sun et al., 2009). Therefore, synapses are considered crucial for the developmental function of the nervous system (Bae & Kim, 2017; Batool et al., 2019; Selkoe, 2002; Skaper et al., 2017). Recent studies demonstrated that a range of neurological diseases could be characterized by the pathological changes in synapses, including abnormal dendritic spine morphology, synaptic loss, and destruction of synaptic plasticity (Zhang, Ben Zablah, et al., 2021). The synapse density can be reduced by 15%–35%, while the loss of synapses in the hippocampus of AD patients can reach as high as 44%–55% (Skaper et al., 2017).

Acrolein, a highly reactive α, β—unsaturated aldehyde is not only an intermediate product derived from lipid peroxidation, but also a common contaminant in diet and environmental pollutant (Igarashi et al., 2018). Some studies found that acrolein could co‐locate with more than 50% of the neurofibrillary tangles and synapses around amyloid protein β (Aβ) in AD patients (Mizoi et al., 2014). Compared with normal subjects, the levels of acrolein in brain tissues, cerebrospinal fluid, and plasma were found to be significantly higher in those patients with mild cognitive impairment (MCI) and AD (Lovell et al., 2001). Moreover, the levels of acrolein and its metabolites could be used to assess the severity of dementia (Igarashi et al., 2015). Acrolein is closely related to synaptic dysfunction (Bradley et al., 2010; Dang et al., 2010). Previously, our studies have been conducted to confirm that acrolein could induce AD‐like pathological changes both in vitro *and* in vivo. The chronic administration of acrolein (2.5 mg/kg, 8 w) can result in mild cognitive impairment and hippocampal neuronal atrophy for SD rats (Huang et al., 2013). Further, we have demonstrated that acrolein could induce synaptic dysfunction (Chen, Lu, et al., 2021). Rho‐associated coiled‐coil‐containing protein kinase (ROCK) can regulate morphogenesis and synaptic plasticity of neuronal dendritic spines in the brain (Magalhaes et al., 2021). However, the underlying mechanism(s) of acrolein‐induced synaptic impairment is still unknown.

In this study, we proved that acrolein‐induced synaptic dysfunction may result from the activation of the RhoA/ROCK2 signaling pathway. ROCK inhibitors or knockdown of ROCK2 could attenuate the synaptic dysfunction and cognitive impairment induced by acrolein. These results demonstrate that the RhoA/ROCK2 signaling pathway plays an important role in acrolein‐induced AD synaptic dysfunction. Therefore, inhibiting the abnormal activation of ROCK2 and improving the synaptic dysfunction induced by acrolein may provide new solutions for the treatment of early AD.

## 2 RESULTS

### 2.1 Acrolein induces axonal rupture and AD‐like pathology but does not affect cell survival in vitro

**FIGURE 1:**
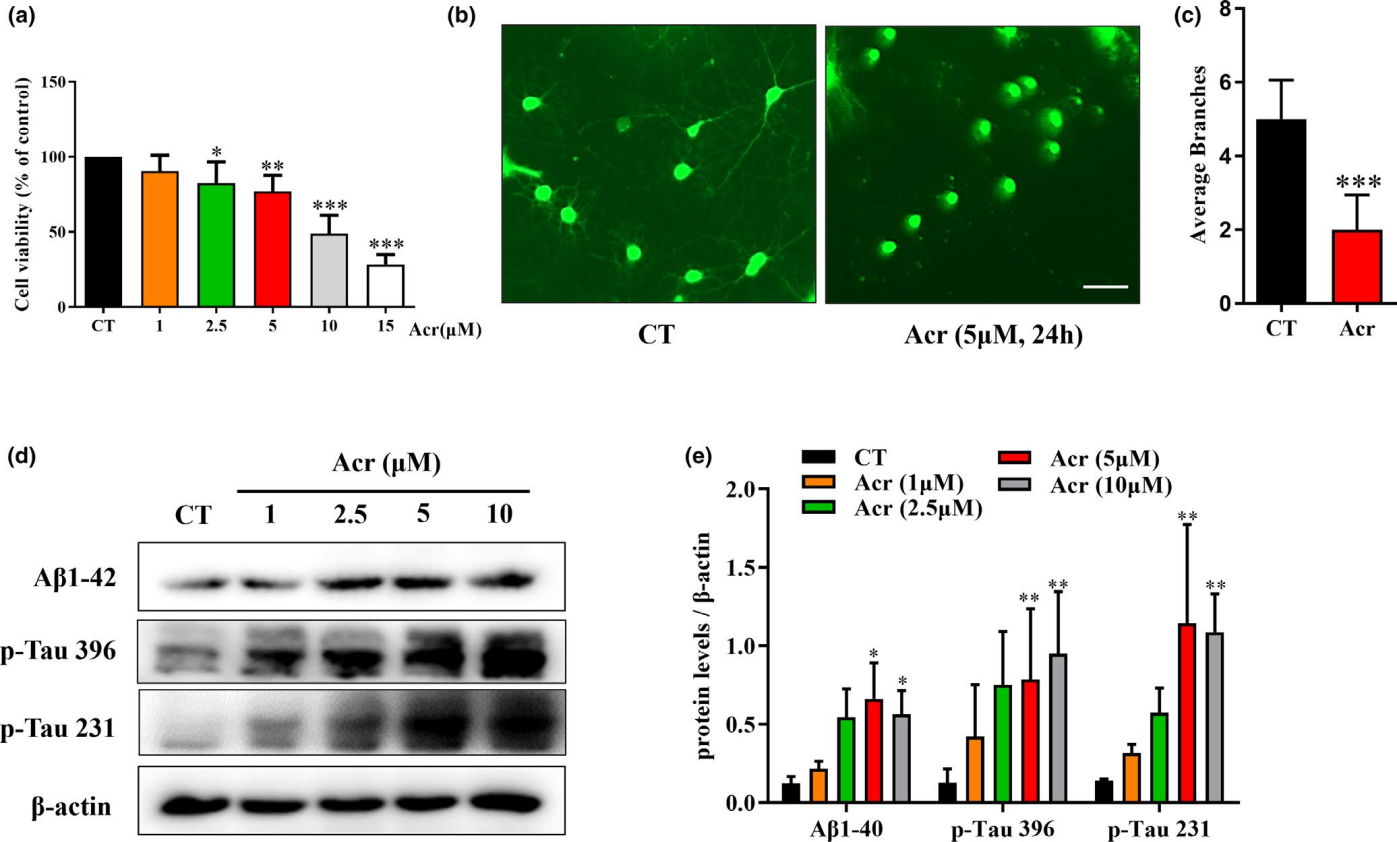
Acrolein induces axonal rupture and increases AD‐like pathological biomarkers but does not affect cell survival in cultured primary cortical. Primary cultured rat cortical neurons were treated with acrolein (5 μM) for 24 h. (a) The effects of acrolein on cell viability in primary cortex neurons at different concentrations for 24 h detected by MTT method. (b) The effects of acrolein on the viability of the primary cortex neurons by Calcien AM staining. Primary cultured rat cortical neurons were treated at DIV7 in the presence of acrolein (1–10 μM) for 24 h. (c) Low concentration of acrolein decreased the numbers of dendrites. (d–e) The cells were then to collected using Western blot methods to analyze the protein levels change of Aβ1‐42, p‐Tau396, p‐Tau231, which reflected AD pathological changes. Scale bar = 40 μm. The data were expressed as mean ± *SD*, *n* = 3. ^*^ *p* < 0.05, ^**^ *p* < 0.01, ^***^ *p* < 0.001 vs. control group

Acrolein could induce cell death in cultured primary hippocampal neurons in a time‐ and concentration‐dependent manner (Lovell et al., 2001), and induced axonal rupture and synaptic dysfunction in cultured primary cortical neurons (Chen, Lu, et al., 2021). The incubation of high levels of acrolein (10–15 μM) for 24 h did induce the cell death of primary cultured cortical neurons (Figure 1a), while low concentrations of acrolein (1–5 μM) caused axonal rupture but not cell death (Figure 1a–c). In addition, we found that 5 μM acrolein can destroy the structure of neuron axons and significantly reduce the number of neurite (Figure S1). Since the concentration of acrolein in the brain of early AD patients is about 4–5 μM (Bradley et al., 2010), 5μM acrolein was chosen for the following studies. We previously reported acrolein could induce AD‐like pathologies in mice (Chen, Lu, et al., 2021), the expression of typical biomarkers of AD was further tested after the treatment of acrolein in vitro. The levels of Aβ1‐42, p‐Tau 396, and p‐Tau 231 were significantly increased in a concentration‐dependent manner in cultured primary cortical neurons. (Figure 1d, e).

### 2.2 Acrolein reduced the number of dendritic spines and synaptic dysfunction in mice

**FIGURE 2:**
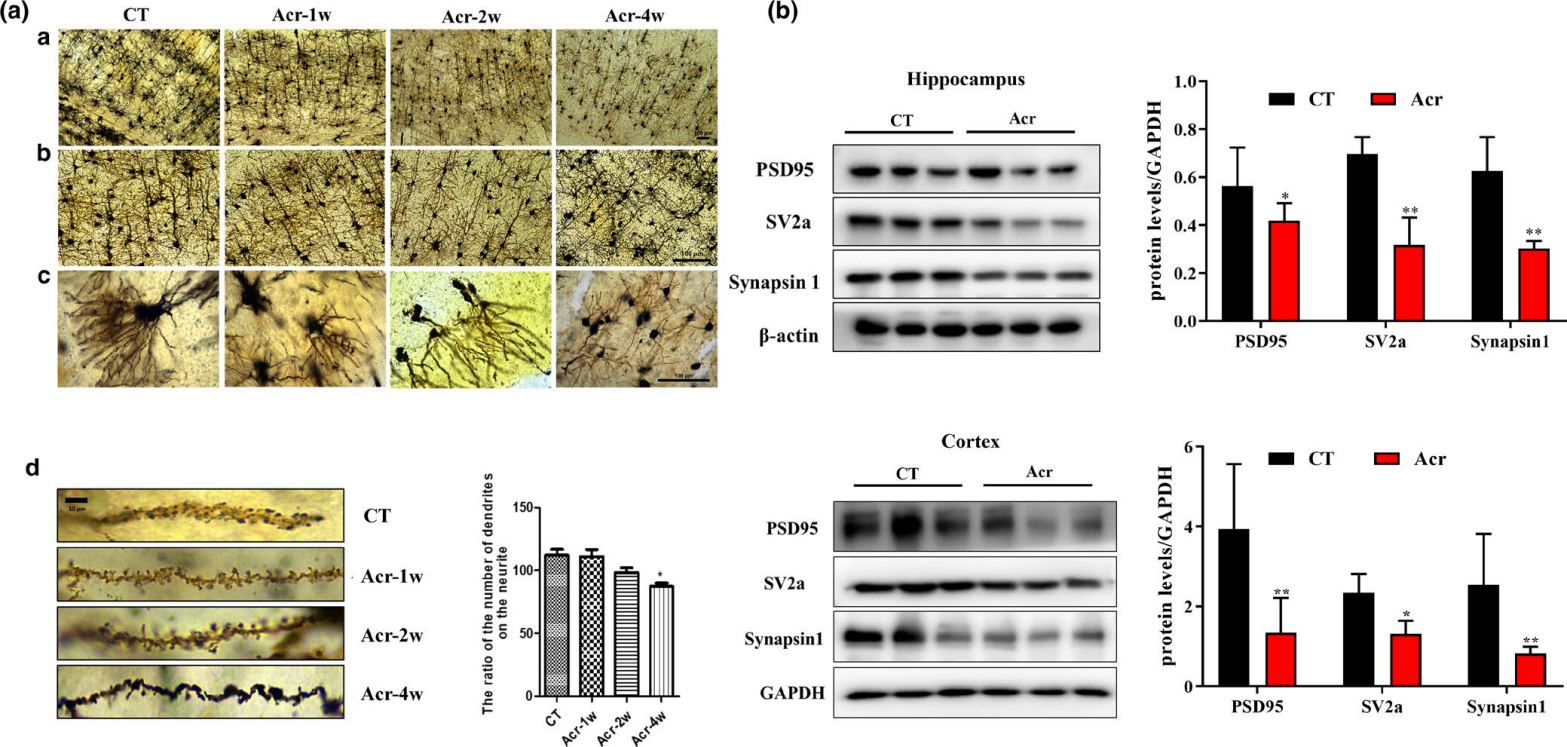
Acrolein induced synaptic rupture in vivo by using Golgi‐Cox staining. The mice were treated with acrolein (3.0 mg/kg/day) or with distilled water for 1–8 weeks. After all behavior tests, the mice were scarified and their brain tissues were harvested for Golgi‐Cox staining and Western blot assay. (A) (a–c) The photographs of synapses in hippocampus and cortex were taken by phase contrast Microscopy, 10× (a), 20× (b), 40× (c) and 100× (d), scale bar = 20 μM; (d) Quantitative analysis of the dendrites. (B) Representative images of proteins reflecting synaptic functional expression (PSD95, SV2a, Synapsin1) in the hippocampus and cortex using Western blot analysis. The data were expressed as mean ± *SEM*, *n* = 3 ^*^ *p* < 0.05, ^**^ *p* < 0.01 vs. control group

It's well known that synaptic dysfunction plays important roles in AD and acrolein can induce axonal rupture and neurites withdraw in cultured neurons (Figure 1b and S1). To verify the effects of acrolein on synapses in vivo, we used Golgi‐Cox staining to detect the number and morphological changes of dendrites in the cerebral cortex of acrolein‐induced mice. Our results showed that acrolein significantly reduced the number of dendritic spines of cortical neurons after 4 weeks of administration, and the dendritic spines were swollen and dysplasia (Figure 2a). Synapsin 1 and postsynaptic density 95 (PSD95) reflect the related functions of pre‐ and post‐synapses, respectively (Zhang, Wu, et al., 2021), while Synaptic Vesicle Glycoprotein 2A (SV2A) is a key protein that reflects the function of synaptic transmission (Zheng et al., 2021). The expression of these synapse‐related proteins in the hippocampus (PSD95: *p *< 0.05; SV2a and Synapsin1: *p *< 0.01) and cortex (SV2A: *p *< 0.05; PSD95 and Synapsin1: *p *< 0.01) of mice was significantly down‐regulated after administration of acrolein for 4 weeks (Figure 2b). Consistent with the results in vitro, the levels of Aβ1‐42 and p‐Tau proteins in the hippocampus (p‐Tau 396 and 231: *p *< 0.05; Aβ1‐42: *p *< 0.01) and cortex (Aβ1‐42, p‐Tau 396 and 231: *p *< 0.05) of acrolein‐treated mice were also significantly increased (Figure S2).

### 2.3 Changes of gene expression profile of acrolein in primary cortical neurons

**FIGURE 3:**
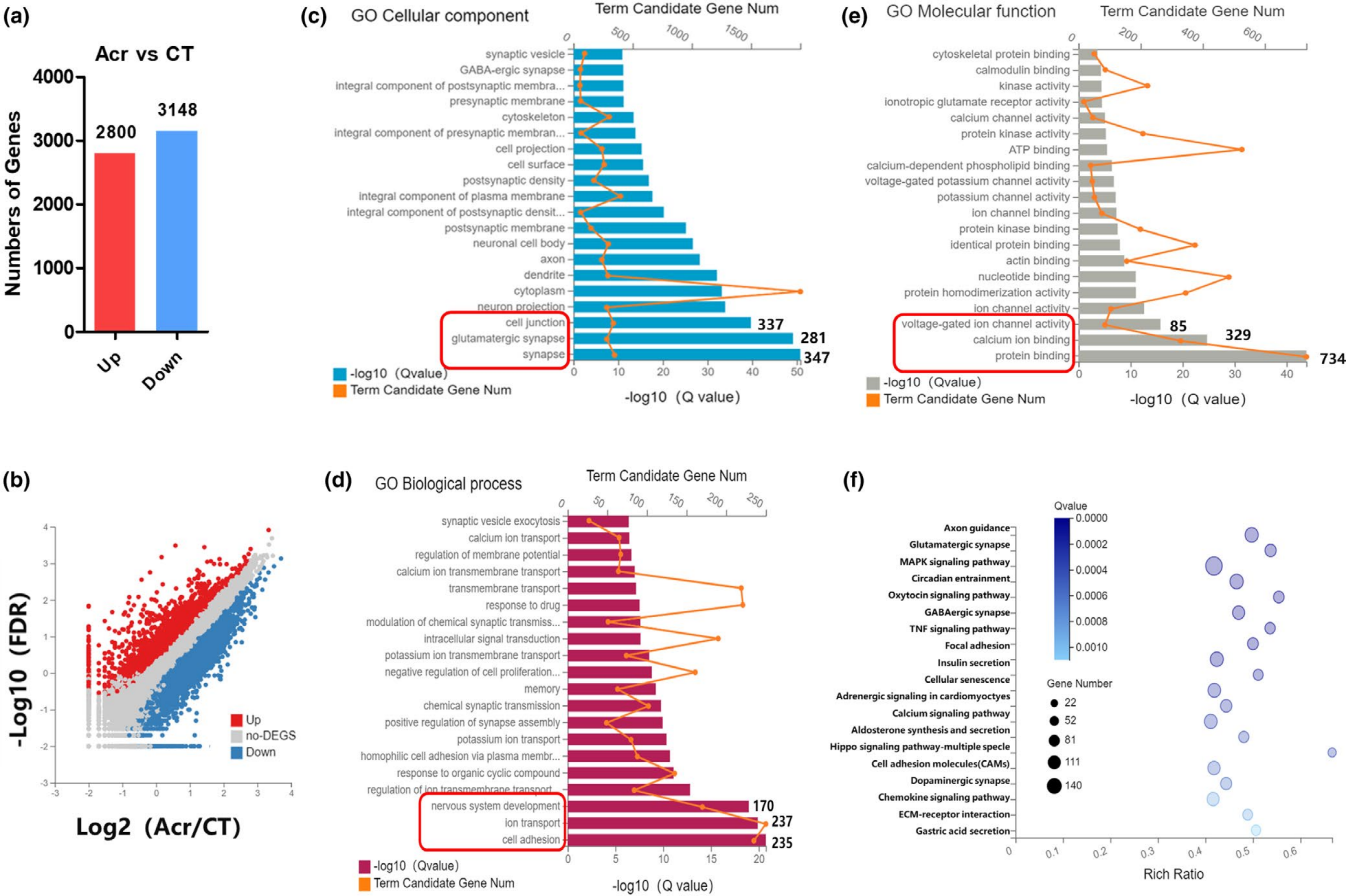
Identification of gene expression profile of acrolein in primary cortical neurons by RNA‐Seq. Primary cultured rat cortical neurons were treated with acrolein (5 μM) for 24 h. (a and b) The numbers of DEGs were represented by bar graph and Volcanic scatter plot. (c) The result of GO cellular component analysis. (d) The result of GO biological pathway analysis. (e) The result of GO molecular function analysis. (f) Visual analysis of enrichment pathway of DEMs in KEGG pathway

To further explore the potential molecular mechanism(s) of acrolein‐induced synaptic damage, we performed RNA sequencing (RNA‐Seq) of primary cortical neurons after acrolein administration. We compared the differential expressed genes (DEGs) between untreated and treated with acrolein (5 μM, 24 h) on primary cortical neurons under the conditions of *p *< 0.05 and fold change>2. Comparing with the control group, acrolein up‐regulated 2,800 genes and down‐regulated 3148 genes (Figure 3a, b). We further performed Gene Ontology (GO) and Kyoto Encyclopedia of Genes and Genomes (KEGG) to enrichmently analyze DEGs. We found that the DEGs caused by acrolein are mainly enriched in "synapses," "glutamatergic synapses," and "cell connections" (Figure 3c). Acrolein interferes with molecular functions such as "protein binding," "calcium ion binding," and "voltage‐gated ion channel activity"(Figure 3d). In addition, acrolein could significantly affect biological processes such as "cell adhesion" and "ion transport"(Figure 3e). Moreover, acrolein could significantly impact the KEGG pathway of “axon guidance process” and “glutamate synaptic” (Figure 3f), further confirming that acrolein interferes with synaptic function. The key DEGs caused by acrolein in these two pathways were marked in Figure S3 and were significantly enriched in the small GTPase family (up‐regulate ROCK, down‐regulate Rac, RasGAP), which is similar to the results of our previous studies about acrolein‐conjugated proteomics (Chen, Chen, et al., 2021).

### 2.4 Acrolein activates the RhoA/ROCK2/p‐cofilin pathway in vivo and in vitro

**FIGURE 4:**
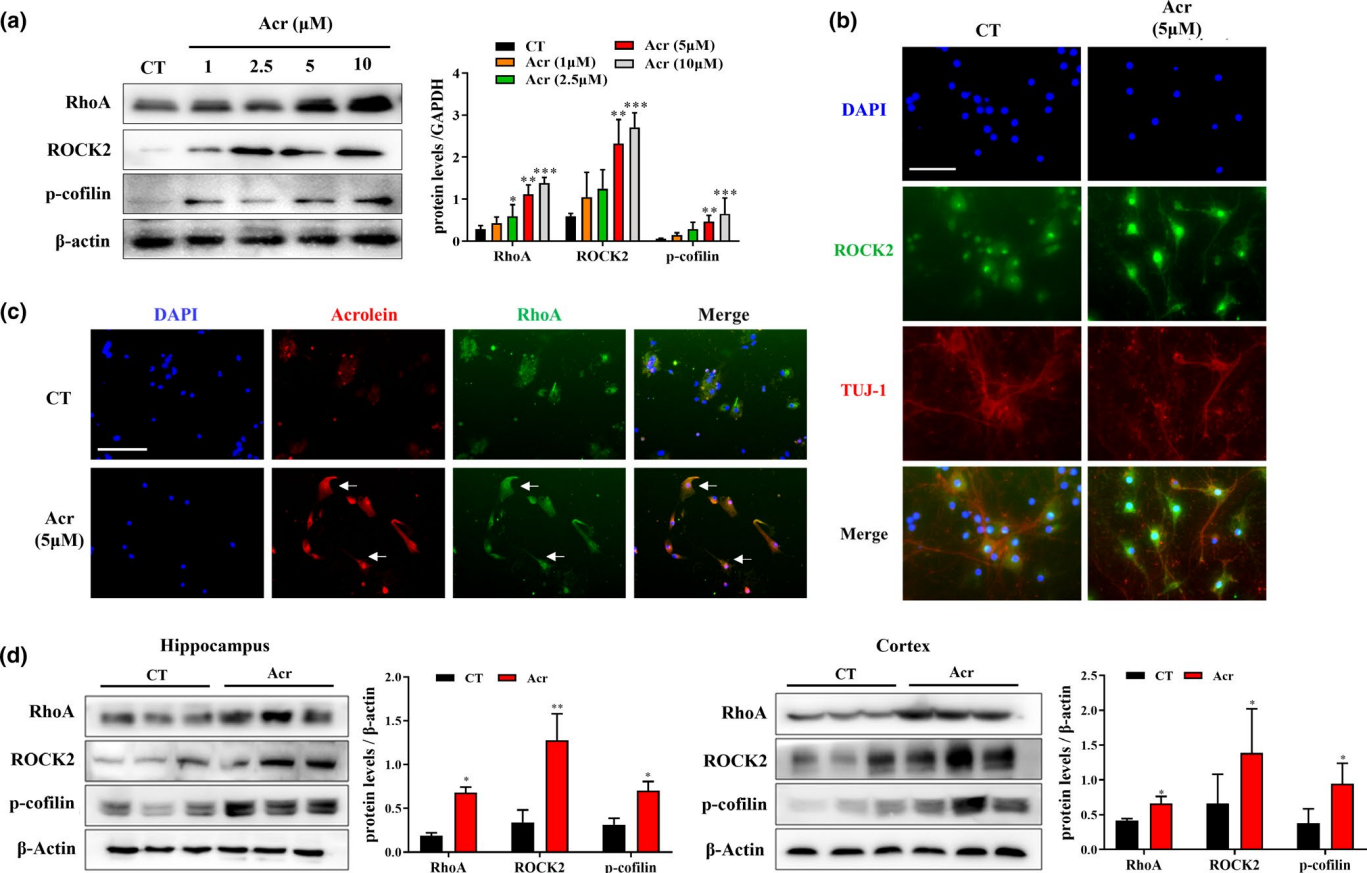
Acrolein could activate the RhoA/ROCK2/p‐cofilin pathway in primary rat cortical neurons and C57BL/6 mice. (a) Primary cultured rat cortical neurons were treated with acrolein (1–10 μM) for 24 h. Cells were then to collected using Western blot methods to analyze the change of protein (RhoA, ROCK2, p‐cofilin) levels. (b) Primary cultured rat cortical neurons were treated with acrolein (5 μM) for 24 h. Cells were then subjected to immunofluorescence analysis for the expression of Tuj‐1 (red) and ROCK2 (green). Nuclei were counterstained with DAPI (blue). (c) primary rat cortical neurons were treated with acrolein (5 μM) for 24 h. Cells were then subjected to immunofluorescence analysis for the expression of acrolein (red) and RhoA (green). Nuclei were counterstained with DAPI (blue). (d) The mice were treated with acrolein (3.0 mg/kg/d) or with distilled water for 4 weeks. The mice were scarified and their hippocampus and cortex tissues were harvested for Western Blotting analysis. Representative images of proteins reflecting synaptic function (RhoA, ROCK2, p‐cofilin) in the hippocampus and cortex by Western blotting analysis. Scale bar = 100 μm. The data were expressed as mean ± *SEM*, *n* = 3. ^*^ *p* < 0.05, ^**^ *p* < 0.01, ^***^ *p* < 0.001 vs. control group

The serine/threonine protein kinase of ROCK includes two isoforms, ROCK1 and ROCK2. ROCK1 is mainly distributed in peripheral tissues such as the lung, liver, and spleen, and ROCK2 is mainly distributed in the heart and nervous system (Yan et al., 2019). Recently, a study reported that ROCK1 mediates the migration of glial cells promoted by acrolein (Fukutsu et al., 2021). Although RNA‐Seq results and our previous acrolein‐conjucated proteomics data indicated that ROCK pathway might be involved in acrolein toxicity. However, it is unknown the exact influence of acrolein on ROCK pathway. Therefore, to verify the effects of acrolein on ROCK pathway in the nervous system, we investigated the effect of acrolein on the RhoA/ROCK2/p‐cofilin pathway on cultured primary cortical neurons. Our results showed that acrolein could increase the protein levels of RhoA, ROCK2, and p‐cofilin in a concentration‐dependent manner (Figure 4a), which was further confirmed in immunostaining of ROCK2 (Figure 4b). The results of immunofluorescence showed that there was co‐localization between acrolein binding protein and RhoA (Figure 4c), which indicated that acrolein may lead to significant activation and triggering a series of synaptic damage effects. Similarly, the levels of RhoA, ROCK2, and p‐cofilin were also significantly increased in the hippocampus (RhoA and p‐cofilin: *p *< 0.05; ROCK2: *p *< 0.01) and cortex (SV2A, RhoA, and p‐cofilin *p *< 0.05) of acrolein‐treated mice (Figure 4d).

### 2.5 ROCK inhibitors can attenuate acrolein‐induced synaptic injuries in vitro

**FIGURE 5:**
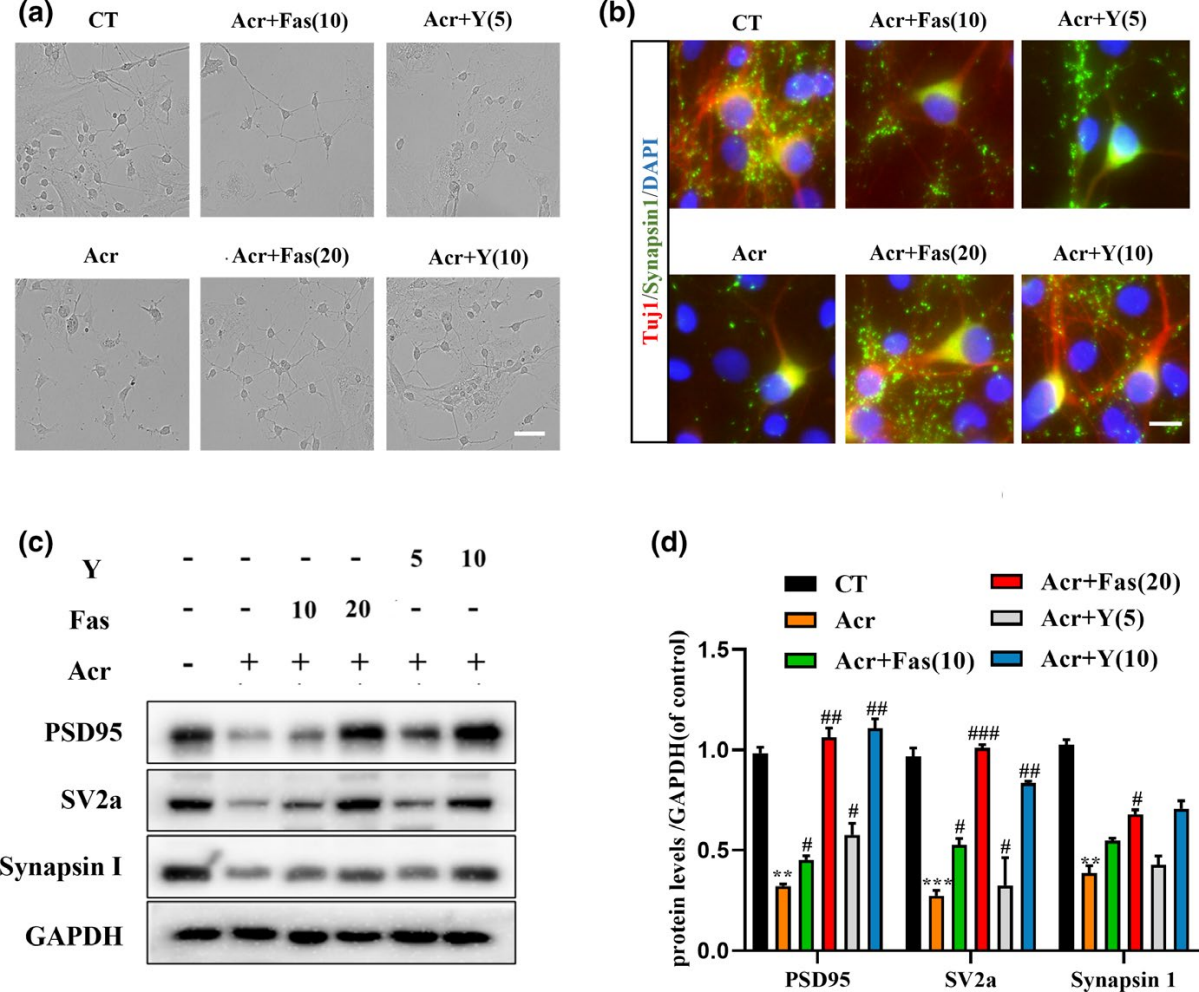
ROCK inhibitors Fasudil and Y27632 can attenuate synaptic disrupter caused by acrolein. Primary cultured rat cortical neurons were treated with acrolein (5 μM) for 24 h, and drug treatment administration‐Fasudil (Fas) 10 Μm/20 μM and Y27632 (Y) 5 μM/10 μM). (a) Cells were analyzed the changes of neuron branches using a phase difference microscope. (b) Cells were then subjected to immunofluorescence analysis for the expression of TUJ‐1 (red) and Synapsin1 (green). Nuclei were counterstained with DAPI (blue). Scale bar = 40 μm. (c) Cells were then to collected using Western blot to analyze the protein levels change of PSD95, SV2a, and Synapsin 1. Acr, Acrolein; Fas, Fasudil; Y, Y27632. The data were expressed as mean ± *SEM*, *n* = 3, **p < 0.01, ***p < 0.001 vs. control group. ^#^ *p* < 0.05, ^##^ *p* < 0.01, ^###^ *p* < 0.001 vs. model group

To further confirm that the roles of the activating RhoA/ROCK2 pathway in acrolein‐induced synaptic damage, two ROCK inhibitors, Fasudil (Fas) and Y27632 (Y), with different chemical structures were evaluated their effects on acrolein‐induced synaptic damage in cultured primary cortex cortical neurons. Both Fasudil (10 and 20 μM) and Y27632 (5 and 10 μM) could dramatically attenuate acrolein‐induced axonal rupture and dendritic spine reduction in a concentration‐dependent manner (Figure 5a). Moreover, Western blot and immunofluorescence results showed that ROCK inhibitors could attenuate the acrolein‐induced decline in synaptic‐related proteins (PSD95: Fas20 and Y10, *p *< 0.01; SV2a: Fas20, *p *< 0.001, Y10, *p *< 0.01; Synapsin 1: Fas20, *p *< 0.05) (Figure 5b, c).

### 2.6 Fasudil improves acrolein‐induced learning and memory impairment in vivo

**FIGURE 6:**
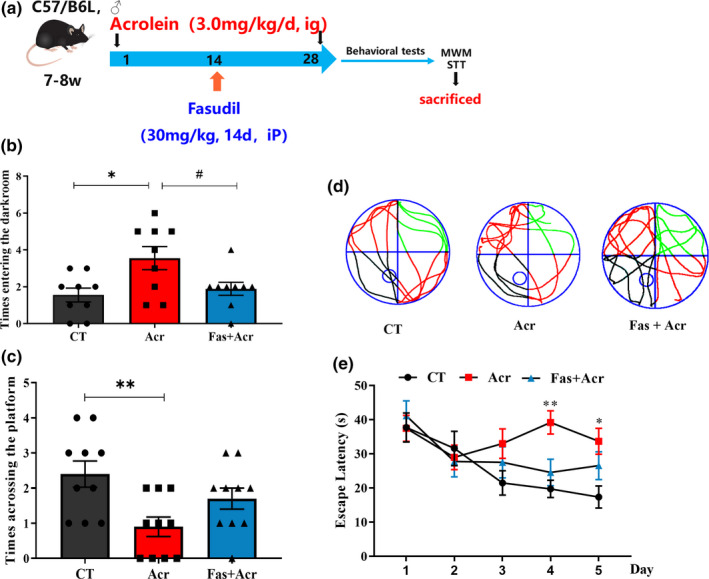
Fasudil improves learning and memory impairment in acrolein‐induced mouse sAD model through step through test and Morris water maze test. The Mice were treated with acrolein (3.0 mg/kg/day) or with distilled water for 4 weeks. After 2 weeks, the treatment group was given fasudil (30 mg/kg, intraperitoneal injection) for another 2 weeks. (a) Scheme of acrolein‐induced mice treated with Fasudil. (b) The times entering the dark room of the mice in the Step‐through test. (c) The swimming tracks of the mice in the probe tests. The circle in the third quadrant represents the location of the hidden platform, while the curves indicate the different swimming strategies of the mice. (d–e) The total and central distance crossing platform of the mice in the probe test. Mean escape latencies of the mice in the hidden platform tests, which were conducted for five consecutive days. Data were presented as the mean ± *SEM*. and analyzed using one‐way ANOVA followed by Tukey post hoc test. *n* = 9–12. *p < 0.05, **p < 0.01 vs. control group

Acrolein induce cognitive impairment in mice and rats (Chen, Lu, et al., 2021; Huang et al., 2013). The firstly marketed ROCK inhibitor Fasudil was evidenced its benefit in mouse models of diverse diseases such as glaucoma, hypertension, and stroke (Chen et al., 2013). Whether should Fasudil be benefit to acrolein‐induced cognitive impairment? Here, C57BL/6 mice exposed to acrolein were treated with or without Fasudil (30mg/kg) for 14 days (Figure 6a). Passive avoidance is a learning task based on associative emotional learning, which is used to determine the short‐term working memory (Anagnostaras et al., 2001). Acrolein‐treated mice presented contextual cognitive deficits compared with WT group due to more times of mistakes that mice enter the dark room, which was attenuated by Fasudil (*p *< 0.05) (Figure 6b). The Morris water maze is mainly used to test the learning and memory ability of mice in spatial positioning. After 5 days of training, all experimental groups presented curves with progressively shorter path length on consecutive days, and Fasudil could significantly shorten the escape latency (Day4: *p *< 0.01; Day5: *p *< 0.05) (Figure 6e). In the spatial probe test on Day 7, the escape platform was removed from the third quadrant. The times of crossing the platform in the target quadrant of acrolein model mice was reduced, while was improved by Fasudil (Figure 6c, d).

### 2.7 Fasudil can improve the acrolein‐induced synaptic function damage and morphology in vivo.

**FIGURE 7:**
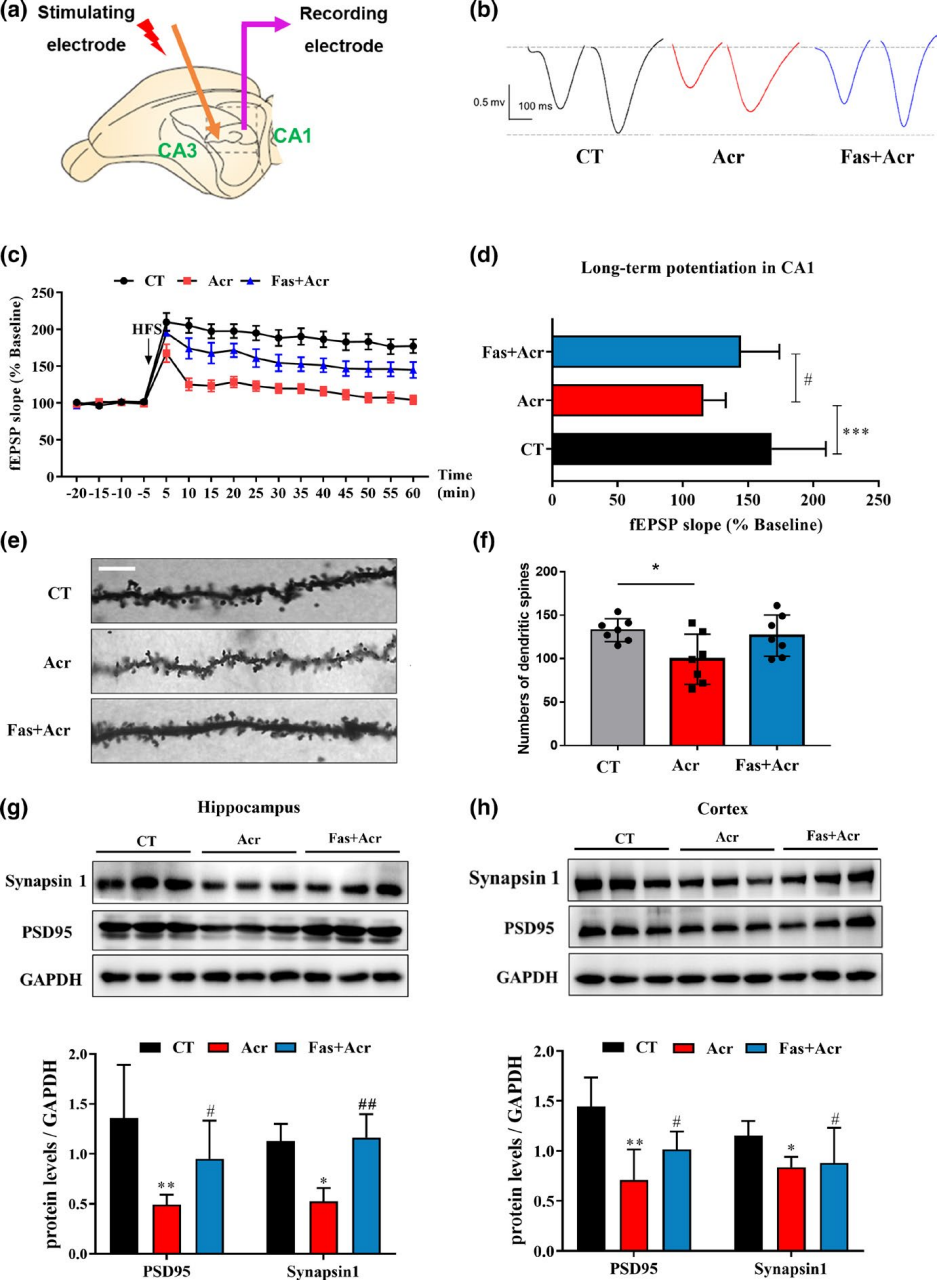
Fasudil can attenuate the synaptic dysfunction induced by acrolein. (a) Diagram of electrophysiological recordings at CA3‐CA1 pathway. (b) Typical average fields potentials before and after Fasudil treatment. (c) Normalized fEPSP slope before and after LTP induction (100 Hz, 50 ms) in control, model, and treatment group. (d) Summary of changes in the average EPSC amplitude 25–30 min after LTP induction. (e–f) The numbers of dendritic spines in DG/CA1 neurons of hippocampus were showed. The photographs were taken by phase contrast microscopy in 100×, scale bar = 10 μm; Quantitative analysis of the spine density. (g) Representative images of proteins reflecting synaptic functional expression (PSD95, Synapsin1) in the hippocampus using Western blot analysis. Data were presented as the mean ± *SEM*. *p < 0.05, **p < 0.01, and ***p < 0.001 vs. control group. *n* = 5. ^#^ *p* < 0.05, ^##^ *p* < 0.01 vs. model group

Cognitive impairment and learning ability of the brain are directly linked to synaptic plasticity as measured in changes of LTP (Malenka & Bear, 2004). We speculated that acrolein can lower LTP in the hippocampus of rat. LTP of hippocampus (CA3‐CA1) in three different rats after the acrolein administration were recorded (Figure 7a, b). The fEPSP, reflecting strengthened synaptic transmission, is usually used to measure the degree of LTP (Tan et al., 2022). Compared with the control group, the LTP of the acrolein group decreased significantly indicating that the LTP of the collateral‐CA1 synapses of the model group was damaged. Fasudil group could rescue these acrolein‐induced decrease (*p *< 0.05) (Figure 7b–d). Through the Golgi‐Cox staining, we observed that the remarkable swollen and broken dendritic spines of hippocampal CA1 pyramidal neurons and granular neurons in the DG area of acrolein group, while these changes were significantly attenuated by Fasudil (Figure 7e, f). Moreover, Fasudil treatment could also significantly attenuate the down‐regulation of synaptic function proteins of Synapsin 1 and PSD95 induced by acrolein in hippocampus (PSD95: *p *< 0.05; Synapsin1: *p *< 0.01) and cortex (PSD95 and Synapsin1: *p *< 0.05) (Figure 7g, h).

### 2.8 Knockdown of ROCK2 can alleviate acrolein‐induced memory impairment and synaptic damage in vivo.

**FIGURE 8:**
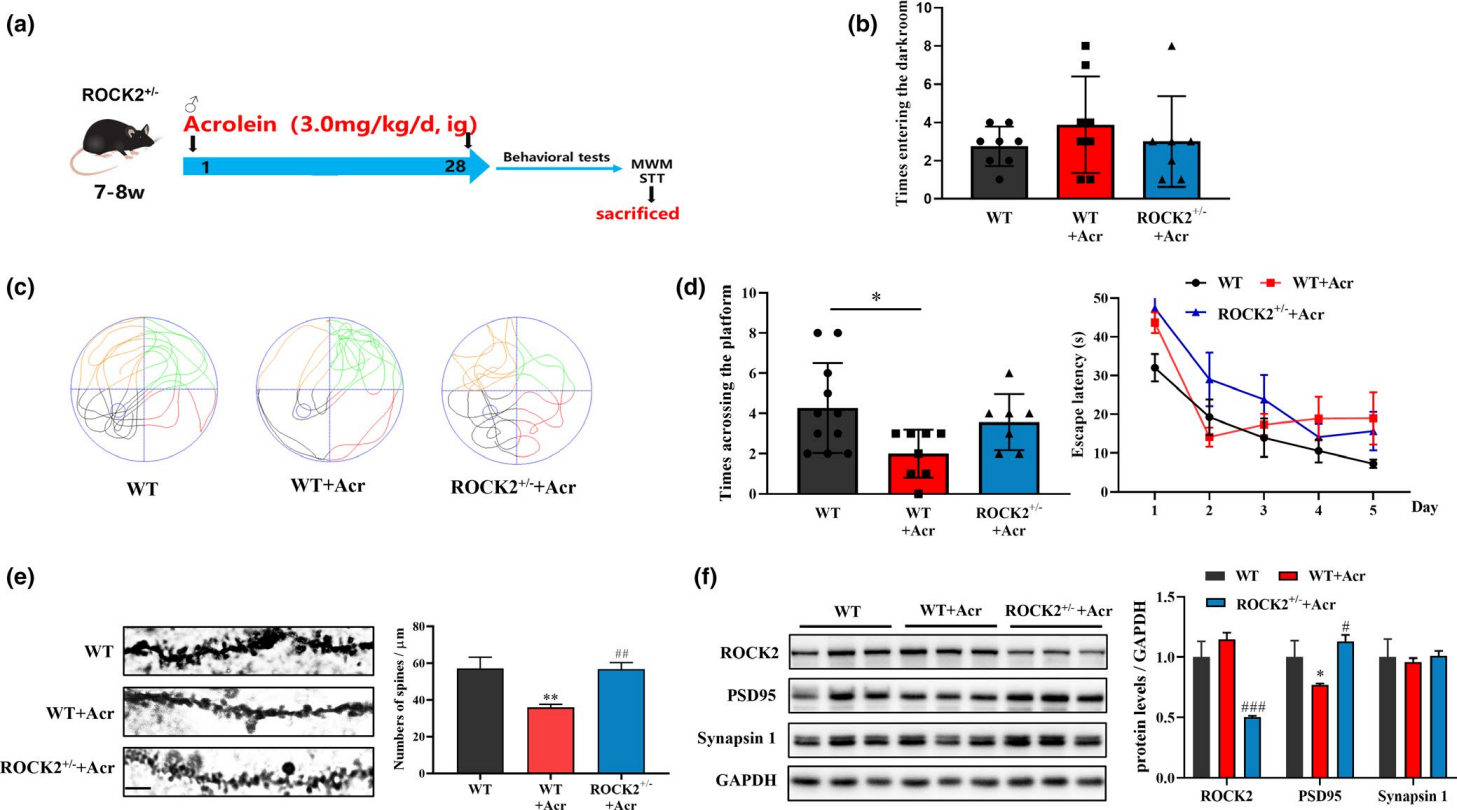
Knockdown of ROCK2 can improve learning and memory impairment and synaptic dysfunction induced by acrolein. The C57B6/L Mice were treated with acrolein (3.0 mg/kg/day) or with distilled water for 4 weeks. The ROCK2 knockdown mice were treated with acrolein (3.0 mg/kg/day) for 4 weeks. (a) Scheme of ROCK2 knockdown mice induced by acrolein. (b) The times entering the dark room of the mice in the step‐through test. (c–d) The swimming tracks of the mice in the probe test of the Morris water maze test. The circle in the third quadrant represents the location of the hidden platform, while the curves indicate the different swimming strategies of the mice; The number of times the mouse passed through the circle was recorded in 60 s. Mean escape latencies of the mice in the place navigation test, which were conducted for five consecutive days. (e) The numbers of dendritic spines in DG/CA1 neurons of hippocampus were showed scale bar = 10 μm; Quantitative analysis of the spine density. (f) Representative images of proteins reflecting synaptic functional expression (ROCK2, PSD95, Synapsin1) in the hippocampus using Western blot analysis. Data were presented as the mean ± *SEM*, *n* = 5–6. *p < 0.05 vs. control group, ^###^ *p* < 0.001 vs. model group

To avoid small molecule inhibitor's off‐target effects, genetically knocking out ROCK2 should be a good way to further verify the role of ROCK2 in acrolein‐induced cognitive impairment. Unfortunately, ROCK2 knockout mice will die at their embryo (Kamijo et al., 2011). So, genetically knocking down mice, ROCK2^+/−^ mice were used to investigate the role of ROCK2 in acrolein‐induced memory impairment and synaptic damage (Figure 8a). In the step‐through test, the ROCK2^+/−^ mice shown the reduced the times of mistakes when mice enter the dark room (Figure 8b). In the Morris water maze test, the times of crossing the platform in the target quadrant of acrolein model mice were significantly decreased, while these changes were attenuated in ROCK2^+/−^ mice (Figure 8c, d). Furthermore, the knockdown of ROCK2 could significantly alleviate damage of dendritic spines (Figure 8e) and the down‐regulation of PSD95 (*p *< 0.05) in the hippocampus (Figure 8f) caused by acrolein.

## 3 DISCUSSIONS

Acrolein is an endogenous aldehyde which is also recognized as a ubiquitous pollutant in the diet and the environment. The levels of acrolein binding protein in brain tissue, cerebrospinal fluid and plasma of patients with MCI and AD are significantly increased, which suggested that the levels of acrolein and its metabolites could be used to predict the development of AD (Igarashi et al., 2015; Mizoi et al., 2014). Studies have shown the chronic administration of acrolein (2.5 mg/kg, 8 w) can result in mild cognitive impairment and hippocampal neuronal atrophy in SD rats (Huang et al., 2013) and mice (Chen, Chen, et al., 2021; Chen, Lu, et al., 2021). Here, we for the first time demonstrated that acrolein induces synaptic dysfunction in vitro and in vivo through activating RhoA/ROCK2 pathway. Pharmacological or genetical down‐regulation of ROCK pathway could attenuated these acrolein‐induced synaptic damage and cognitive dysfunction.

In neuroscience, “synaptic plasticity” is the ability of synapses to strengthen or weaken over time, in response to increases or decreases in their activity. It is generally believed that synaptic plasticity is one of the important neurochemical foundations of learning and memory (Sun et al., 2009). Loss of synaptic stability can lead to the destruction of neural circuits and eventually develop into various brain diseases, such as AD, stroke, and autism (Colom‐Cadena et al., 2020; Murphy & Corbett, 2009).

Acrolein is a member of a large class of structurally related chemicals known as the type‐2 alkenes (Lopachin et al., 2007). It has been shown in some studies that acrolein can give rise to the sulfhydryl adducts with some specific amino acid sites of synaptosome‐related proteins in vitro and accumulate in synaptosomes, thus destroying the conformations of membrane proteins and phospholipids in the membranes of synaptosomes. Ultimately, acrolein could destroy the glutamate uptake and glucose transport capacity in the synaptosome (Barber et al., 2007). In vivo and in vitro studies revealed that acrolein could inhibit membrane vesicle fusion proteins, thereby reducing the release of presynaptic neurotransmitters and disrupting the process of neurotransmission. Therefore, the synaptic damage induced by acrolein (Lopachin et al., 2007) in might be the key cause of early AD pathological symptoms. Since the concentration of acrolein in the brain of early AD patients is about 4–5 μM (Bradley et al., 2010; Tsou et al., 2018). We simulated the acrolein concentration (5 μM) in the brains of early AD patients on primary cortical neurons in vitro (Lovell et al., 2001). We observed that low concentration of acrolein (5 μM) cause the rupture of neuronal axons and decrease the number of dendrites but not induced cell death (Figure 1 and S1), as well as down‐regulate the level of related synaptic proteins (Chen, Lu, et al., 2021) (Figure 1). The synapse is the place where Aβ peptides are generated and is the target of the toxic Aβ oligomers. Aβ and abnormal p‐Tau together lead to synaptic deficits in early phases of AD (Pelucchi et al., 2022). We also confirmed that acrolein can up‐regulate Aβ and p‐Tau in vivo and in vitro (Figure 1 and S2). In vivo, acrolein can significantly reduce the number of dendritic spines in the cerebral cortex of mice, resulting in abnormal changes in the morphology of dendritic spines (Figure 2). These results indicated that acrolein can destroy the synaptic structure and cause synaptic damage.

Mounting evidence demonstrated that impaired synaptic function is an early pathological manifestation of AD (Andersen et al., 2021; Blennow & Zetterberg, 2018). The brain tissue and synaptic structure of patients with advanced AD has been irreversibly damaged (Braak & Del Tredici, 2011; Robbins et al., 2021). At the MCI stage, there is no obvious organic disease in the brain of AD patients, and the decline in cognitive level may be caused by abnormal synaptic function. Impairment of synapse function occurs before synapse loss and neuronal degeneration (Budak & Zochowski, 2019). Therefore, research on synaptic damage is particularly critical for the treatment of early AD (Jackson et al., 2019).

RNA sequencing (RNA‐seq) technologies provides a broader picture of the transcriptome, enabling to uncover the mechanism(s) underlying some complex biochemical changes (Sutherland et al., 2011). In this study, we used RNA‐seq to further uncover the mechanism(s) of acrolein‐caused synaptic damage. After enriching the differential genes, we found that acrolein can significantly interfere with the related genes of the small GTPase family, and affect the Wnt signaling pathway and MAPK signaling pathway to affect the axon growth process (Figure 3 and S3). In our previous studies on acrolein‐conjugated proteomics, we found that proteins adducted with acrolein are mainly conjugated with 14–3–3 protein and members of small GTPase family (Chen, Chen, et al., 2021). All proteomics results point to acrolein may abnormally activate the ROCK, which is known as the main downstream effector molecule of Rho in small GTPase family.

ROCK is a serine/threonine kinase and two isoforms, ROCK 1 and ROCK 2, were isolated till now (Cai et al., 2021). ROCK1 is mainly distributed in peripheral tissues such as the heart and skeletal muscle, and ROCK2 is mainly distributed in the nervous system (Yan et al., 2019). After ROCK is activated by Rho, the actin‐binding protein cofilin is abnormally phosphorylate and loses the function of cutting actin (Guiler et al., 2021). ROCKs have more than 30 downstream substrates, the most classic ones include Tau, Limk1, and p‐cofilin, etc (Shi & Wei, 2022). Inhibition of RhoA/ROCK2/Limk1/cofilin signaling pathway rescued the memory impairments and synaptic disorder in AD rat (Han et al., 2020). ROCK inhibitors could also reduce synaptic damage and promote nerve regeneration in AD mice (Yan et al., 2021). Recent studies indicated that ROCK2 is a promising therapeutic target for AD (Cai et al., 2021; Weber & Herskowitz, 2021). Some studies indicated that acrolein is closely related to ROCK1 (Fukutsu et al., 2021) but no relevant biological experiment conducted to substantiate the relationship between acrolein‐induced early AD and ROCK2 in the brain. In this study, acrolein could activate the RhoA/ROCK2/p‐cofilin pathway both in vivo and *vitro* (Figure 4). Taken together, we speculated that acrolein could regulate Aβ metabolism and p‐Tau phosphorylation by activating the ROCK2 pathway, which was consistent with the studies in transgenic AD mice (Herskowitz et al., 2013). These results further support the involvement of acrolein in AD‐like pathology.

Pharmacological strategies are convenient tool to uncover the role of potential signaling pathway. Next, we selected two ROCK inhibitors with different chemical structures, Fasudil and Y27632, and ROCK2 knockdown mice to combat the synaptic damage caused by acrolein. In vitro, the two inhibitors Fasudil and Y27632 attenuated the axonal ruptures and synaptic disruption cause by acrolein (Figure 5). Since Fasudil is a marketed ROCK inhibitor, we used Fasudil to clarify the effect of acrolein on the ROCK2 pathway in vivo (Sellers et al., 2018). Our result confirmed that Fasudil improved acrolein‐induced learning and memory impairment (Figure 6), improved the acrolein‐induced LTP reduce, and restored synaptic plasticity (Figure 7). Because small molecule inhibitors may have off‐target effects. We further used ROCK2 knockdown mice to verify the relationship between ROCK2 and acrolein. Similarly, ROCK2 knockdown mice could partially alleviate acrolein‐induced cognitive impairment and synaptic damage (Figure 8).

It is worthy of pointing out that neither ROCK2 inhibitor nor knockdown of ROCK2 fully blocked the acrolein‐induced cognitive impairment although they have the benefit tendency. The reason might be that acrolein induced mild cognitive impairment, which mimic the early stage of AD (Chen, Lu, et al., 2021) and acrolein is a pollutant that can adducted to a wide range of proteins and then affects multiple pathways beyond of ROCK to induce early AD (Moghe et al., 2015). The omics data demonstrated that acrolein is related to mitochondrial function, endoplasmic reticulum stress, and immune dysfunction (Alfarhan et al., 2020; Chen, Chen, et al., 2021; Moghe et al., 2015). The exact mechanisms of acrolein toxicity are needed to be further explored.

In general, our study confirmed that acrolein induces synaptic dysfunction through activating RhoA/ROCK2 signaling pathway in vitro and in vivo. Acrolein can induce the similar early AD pathology as that in other transgenic mice. In addition, ROCK inhibitors or knocking down ROCK2 could attenuate the cognitive impairment and synaptic dysfunction induced by acrolein, suggesting that ROCK2 may be a potential target for the treatment of early AD. Although more studies are needed, our findings for the first time demonstrated that RhoA/ROCK2 signaling pathway plays a critical role in acrolein‐induced synaptic damage and cognitive dysfunction, suggesting inhibition of ROCK2 should benefit to the early AD.

## 4 MATERIAL and METHODS

### 4.1 Animals

Male C57BL/6 mice, weighing 18−20 g, were housed under a 12‐h light:12‐h dark cycle in a temperature (21 ± 1°C) and humidity‐controlled (65%) environment and were daily operated in parallel. Mice in the acrolein group were orally administered with acrolein (3.0 mg/kg/day) for 4 weeks, the treatment group was given Fasudil (30 mg/kg) by intraperitoneal injection from two weeks, and the other groups with distilled water. Mice in the control (CT) group were of the same age with acrolein group (the age of 12 weeks). All procedures and care of mice conformed to National Institutes of Health guidelines and were approved by Sun Yat‐Sen University (C2018−077DS).

Male Sprague–Dawley (SD) rats, weighing 180−220 g, were housed under a 12‐h light:12‐h dark cycle in a temperature (21 ± 1°C) and humidity‐controlled (65%) environment and were daily operated in parallel. Mice in the acrolein group were orally administered with acrolein (5.0 mg/kg/day) for 4 weeks, the treatment group was given Fasudil (30 mg/kg) by intraperitoneal injection from two weeks, and the other groups with distilled water (Huang et al., 2013). All procedures and care of mice conformed to National Institutes of Health guidelines and were approved by Sun Yat‐Sen University (C2018−077DS).

We thank Dr. William Caldwell (Medical College of Georgia, Augusta University, Augusta, GA, USA), to provide breeding pairs of ROCK2^+/−^ mice (Yan et al., 2019). The mice were housed in Guangzhou University of Traditional Chinese Medicine (NO.00267912). ROCK2 knockdown mice in the acrolein group were orally administered with acrolein (3.0 mg/kg/day) for 4 weeks, and the other groups with distilled water.

### 4.2 Primary neuron culture and drug treatment

Rat hippocampal neurons were prepared from E18 embryos and cultured at high‐density for widefield fluorescence microscopy imaging as previously described (Swanger et al., 2011). Cells were seeded at 2.0 × 10^5^ cells per cm^2^ in DMEM (Sigma) supplemented with 10% (vol/vol) FBS (Gibco) and incubated at 37°C in a humidified 5% CO_2_‐containing atmosphere. At 4 h after plating, the medium was replaced with Neurobasal medium (Invitrogen, Thermo Scientific) supplemented with 2% B27 (Invitrogen) and glutamine (4 mM; Invitrogen). At 8 to 9 d in culture, neurons were under administration of acrolein (1, 2.5, 5, and 10 μM) for 24 h. To inhibit ROCKs, Y‐27632 (merck) and Fasudil (Selleck) were dissolved in DMSO (sigma) and stored at 100 mM. Finally, Y27632 and Fasudil were added to cells at final concentrations of 5, 10, and 10, 20 μM, respectively, (Jeon et al., 2013; Liu et al., 2016). The vehicle control (labeled mock) was Neurobasal for all experiments.

### 4.3 Western blotting

Western blotting was performed as described previously by our laboratory (Chen et al., 2020). Primary antibodies include anti‐Aβ1‐42 (Bioss, BS0107R, 1:500), anti‐p‐Tau396 (Abcam, ab109390, 1:1000),anti‐p‐Tau231 (Abcam, ab151559, 1:1000), anti‐pan‐actin (Thermo‐fisher, MA1‐140, 1:5000), anti‐PSD95 (CST, 3450S, 1:1000), anti‐SV2a (Abcam, ab32942, 1:1000), anti‐Synapsin1 (Abcam, ab52642, 1:1000), anti‐RhoA (Abcam, ab187027, 1:1000), anti‐ROCK2 (Abcam, ab71598, 1:1000), anti‐p‐cofilin(Abcam, ab131274, 1:1000), and anti‐GAPDH (Thermo‐fisher, MA515738, 1:5000).

### 4.4 Immunofluorescence

Immunofluorescence was performed as described previously by our laboratory (Chen et al., 2020). Primary antibodies include anti‐Tuj1 (Biolegend, 801202, 1:500), anti‐Synapsin1 (Abcam, ab52642, 1:500), anti‐RhoA (Abcam, ab187027, 1:100), anti‐ROCK2 (Abcam, ab71598, 1:100), and anti‐acrolein (Abcam, ab240906, 1:200). Cells were incubated with goat‐anti‐rabbit secondary antibodies conjugated to Alexa Fluor 488 (Invitrogen, Carlsbad, CA). The nuclei were incubated with DAPI (Beyotime, China, catalog, C1002, 5 mg/ml in PBS) in the dark. Immunofluorescent images were acquired through a confocal microscope (Olympus FV3000, Japan).

### 4.5 RNA extraction and RNA‐sequencing

Total RNA was extracted by Trizol method. RNA degradation and contamination was monitored on 1% agarose gels. RNA purity was checked using the NanoPhotometer spectrophotometer (IMPLEN, CA, USA). RNA concentration was measured using Qubit RNA Assay Kit in Qubit 2.0 Flurometer (Life Technologies, CA, USA).

RNA‐seq was performed independently and uniformly for each sample. Clean reads were aligned to the reference gene sequence using bowtie‐2, and the gene expression levels of each sample were calculated. DEG detection was conducted using the DEGseq method. The statistical results were based on the MA‐plot method. The numbers of reads of specific genes obtained from the sample were sampled randomly, and the *p*‐values were then calculated according to the normal distribution and corrected to *q*‐values. To improve the accuracy of DEG detection, genes with a difference multiple of >2, and a *q*‐value of ≤0.001 were screened and defined as significantly DEGs.

### 4.6 Golgi‐Cox staining

Golgi‐Cox staining was performed as described previously by our laboratory (Chen, Lu, et al., 2021).

### 4.7 Morris water maze

The Morris water maze was used to test spatial reference learning and memory in all groups (Morris, 1984). The maze was made of black plastic circular tank (120 cm diameter) containing a 10 cm‐diameter hidden platform, which was placed in the target quadrant and submerged 1 cm below the water surface for all trials. Mice received a 1‐day adaptability trial before the hidden platform trial. In the hidden platform trial, mice were placed into every quadrant in sequence to find the hidden platform for 60 s. Mice were allowed to swim until the hidden platform was found within 60 s for 5 days. In the probe trail, the platform was removed and the mice were placed into the tank. Then the number of platform crossing and the time and the percentage of time and path in target quadrant were recorded during 60 s.

### 4.8 Step‐through test

A step‐through passive avoidance apparatus was used in the test, which is consisted of glass box divided into two compartments (dark/light) with identical dimensions. Mice underwent an exploration session with 180 s on the first day and the following day mice were subjected to one training session for 300 s with a foot shock. After 24 h, mice were subjected to memory retention test with no electrical shock for 300 s, and the time of mice to entry the dark chamber (Error frequency) and the time entering the dark chamber (Latency) were recorded.

### 4.9 In vivo electrophysiological recording

Following the procedures described previously (Liu et al., 2017), animals anesthetized with urethane (1.5 g/kg, i.p.) were placed in a stereotaxic frame. fEPSPs were recorded from the stratum radiatum in CA1 following electrical stimulation of the Schaffer collateral–commissural pathway. Electrophysiological criteria were used to determine the optimal electrode placement. The recording electrode was positioned 3.4 mm in rat posterior to bregma, 2.5 mm in rat lateral to midline, and the depth of recording electrode was about 2.2 mm in rat from dura. The stimulating electrode was positioned 4.2 mm in rat posterior to bregma and 3.8 mm lateral to midline, and about 4.7 mm in depth in rat from dura. A single square pulse of voltage at low frequency (0.066 Hz, 0.2 ms duration) was used to evoke fEPSPs and the intensity of the test stimulus was adjusted to produce 50–55% of maximum response. High‐frequency stimulation (HFS, 100 Hz, 50 pulses, four trains at 15 s interval) protocol was used to induce LTP. The intensity of HFS was raised to evoke 75% of maximum fEPSPs amplitude. The amplitudes of fEPSPs were determined on‐line by LTP program (http://www.ltp‐program.com). In each experiment, the responses to twenty consecutive test stimuli were averaged. The mean amplitudes of responses before HFS served as baseline.

To measure the frequency facilitation in CA1 synapses, a presynaptic form of short‐term plasticity, 100 conditioning stimuli at 2, 4, and 8 Hz were in turn delivered in the same animal at 20 min intervals. Stable baseline was recorded before conditioning stimuli. The intensity of conditioning stimuli was identical to test stimuli.

### 4.10 Bioinformatic analysis

The raw data were searched and identified through the UniprotKB (http://www.uniprot.org/). Bioinformatic analysis was integrated on Gene Ontology (GO) categories, Kyoto Encyclopedia of genes and Genomes (KEGG) pathway tool (http://www.genome.jp/kegg/), and STRING (https://www.stringdb.org). The hypergeometric test is statistic method for an enrichment analysis to test whether a GO term and KEGG pathways are statistically enriched for the given set of genes. Use Multiquant software to extract chromatographic peak area and retention time. The standard substance of energy metabolites was used to correct the retention time, and the metabolites were identified.

### 4.11 Statistical analysis

All results were reported as means ± standard deviation (*SD*) or means ± Standard Error of Mean (*SEM*) at least three independent experiments. Differences between groups were tested using one‐way analysis of variance (ANOVA), followed by a Tukey–Kramer test. *p * < 0.05 was considered to indicate a statistically significant difference.

## CONFLICT OF INTEREST

None declared.

## AUTHORS’ CONTRIBUTIONS

**Zeyu Zhu and Junfeng Lu involved in** investigation, writing—original draft, and formal analysis. **Shuyi Wang, Jiakai Pi, Weijia Peng, and Xin Zhou did** investigation. **Yang Yang performed** formal analysis. **Xifei Yang and Wenjun Xin involved in** formal analysis and validation. **Wei Yin and Lin Yao involved in** writing, project administration, and supervision. **Rongbiao Pi involved in** conceptualization, project administration, and supervision. All authors read and approved the final manuscript.

## Supporting information

Fig S1 Acr

Fig S1 CT

Fig S2

FIg S3

Supplementary Material

## Data Availability

The data that support the findings of this study are available from the corresponding author upon reasonable request.
