# Corrigendum to: Acrolein, an endogenous aldehyde induces synaptic dysfunction in vitro and in vivo: Involvement of RhoA/ROCK2 pathway

**DOI:** 10.1111/acel.13945

**Published:** 2023-07-31

Zhu, Z., Lu, J., Wang, S., Peng, W., Yang, Y., Chen, C., Zhou, X., Yang, X., Xin, W., Chen, X., Pi, J., Yin, W., Yao, L., & Pi, R. (2022). Acrolein, an endogenous aldehyde induces synaptic dysfunction in vitro and in vivo: Involvement of RhoA/ROCK2 pathway. *Aging Cell*, 21, e13587. https://doi.org/10.1111/acel.13587

In our above published article, we noticed the following errors in Figures 2 and 4 and in Section 2.6.

1. In Figure 2A, the 20× image in Figure 2b was misaligned with the 10× image in Figure 2a. We replaced the 40× image of acr‐2w to the corresponding 20× image. When reviewing the graph, we found that the purpose of Figure 2c was to reveal the high magnification of synapses in the hippocampus region. In the original text (Figure Legend 2a), we have mentioned the hippocampus and cortex, but there were no detailed markings in Figure 2c and in Figure Legend 2a. At the same time, we checked the data of our works and present the more representative hippocampal Golgi‐staining images in Acr‐4w group. The revised figure along with its caption is shown below

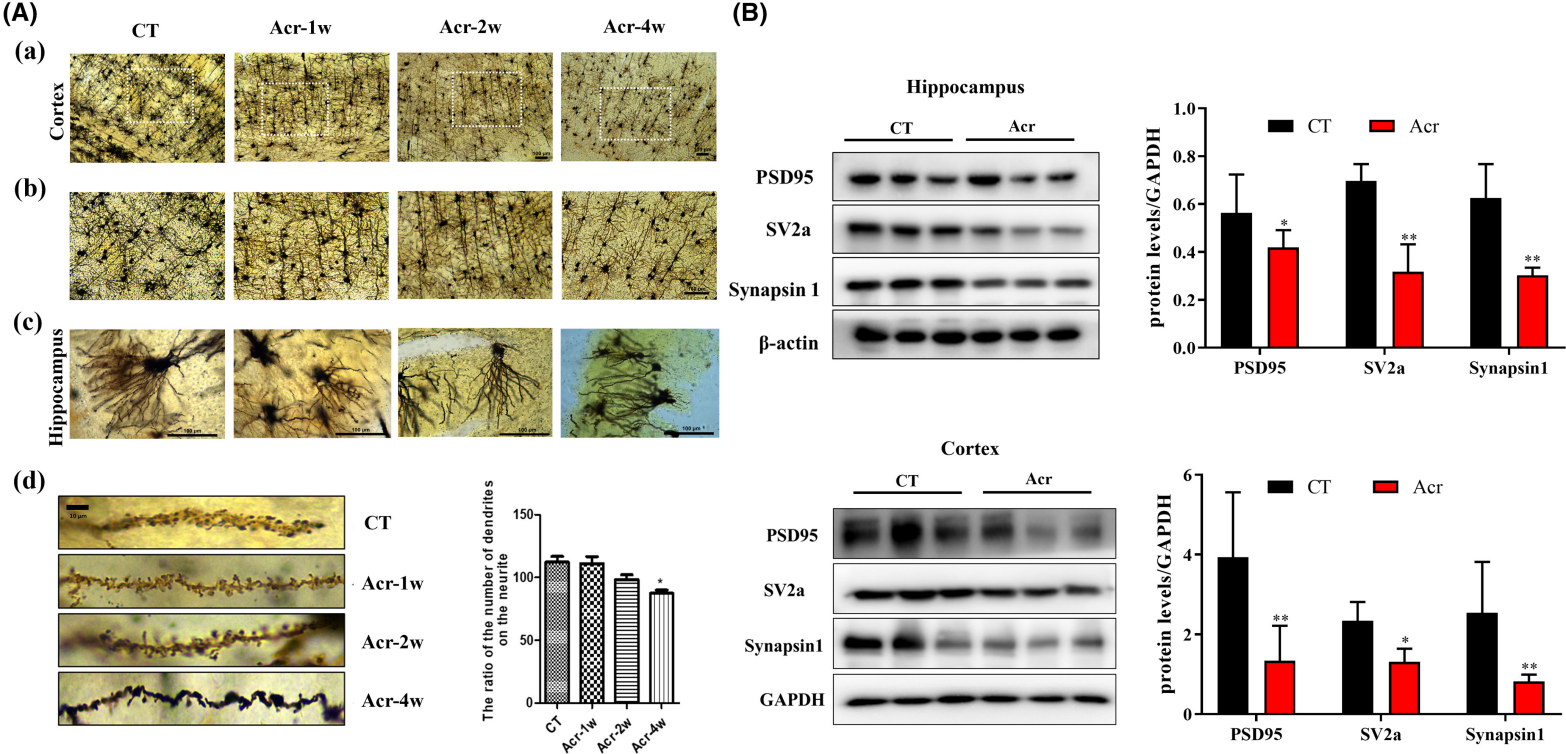

**FIGURE 2**. Acrolein induced synaptic rupture in vivo by using Golgi‐Cox staining. The mice were treated with acrolein (3.0 mg/kg/day) or with distilled water for 1–8 weeks. After all behavior tests, the mice were scarified and their brain tissues were harvested for GolgiCox staining and Western blot assay. (A) (a–c) The photographs of synapses in hippocampus and cortex were taken by phase contrast microscopy, cortex: 10× (a), 20× (b) and hippocampus: 40× (c). (d) Quantitative analysis of the dendrites. (B) Representative images of proteins reflecting synaptic functional expression (PSD95, SV2a, Synapsin1) in the hippocampus and cortex using western blot analysis. The data were expressed as mean ± SEM, *n* = 3 *p < 0.05, **p < 0.01 versus control group.

2 In Figure 4d, the incorrect ROCK2 band has been replaced

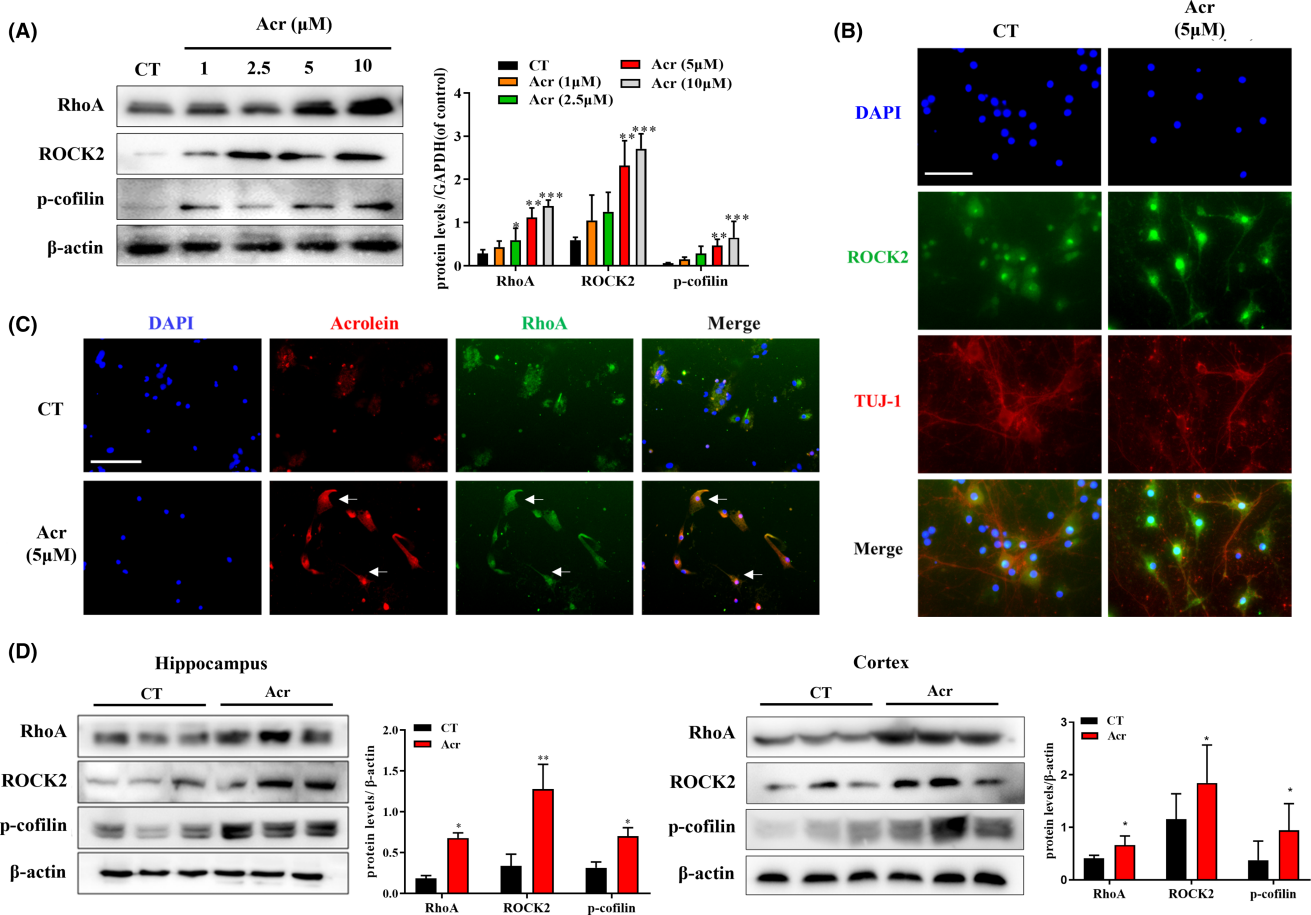

3 In section 2.6, line 8 “Acrolein‐treated mice presented contextual cognitive deficits compared with WT group due to more times of mistakes that mice enter the dark room, which was attenuated by Fasudil (*p* < 0.05) (Figure 6B).” should read as “Acrolein‐treated mice presented contextual cognitive deficits compared with WT group due to more times of mistakes that mice enter the dark room, which was attenuated by Fasudil (*p* < 0.05; Figure 6B, partial data of CT and Acr group cited from C Chen, Lu, et al., 2021).”

We apologize for these errors.

