# Supplementary figures and images for: Acrolein, an endogenous aldehyde induces synaptic dysfunction in vitro and in vivo: Involvement of RhoA/ROCK2 pathway
Source: Aging Cell. 2022 Mar 22;21(4):e13587. doi: 10.1111/acel.13587 (PMC9009232; doi:10.1111/acel.13587)

**A**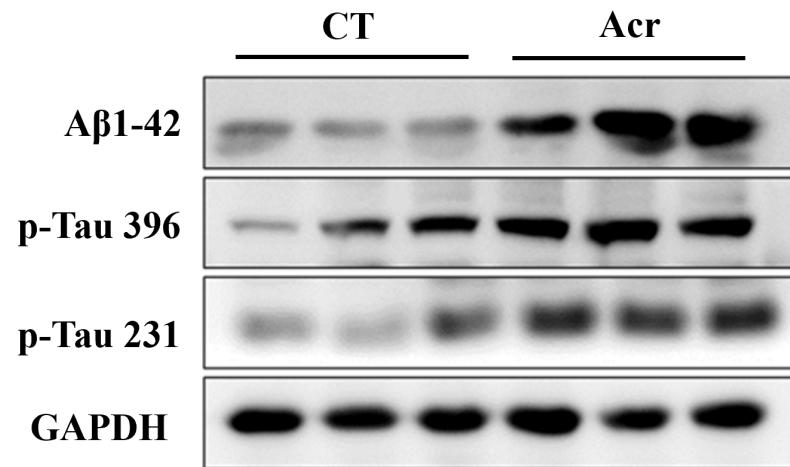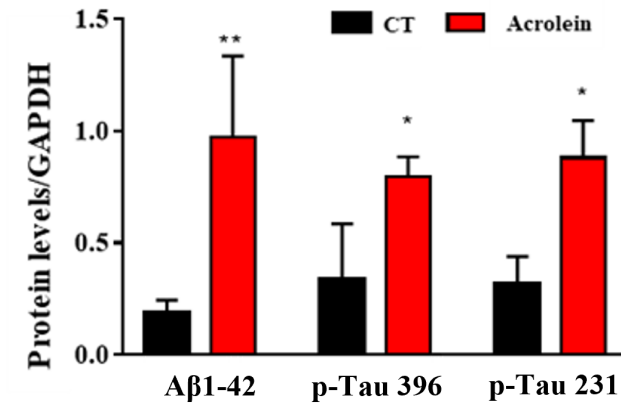**B**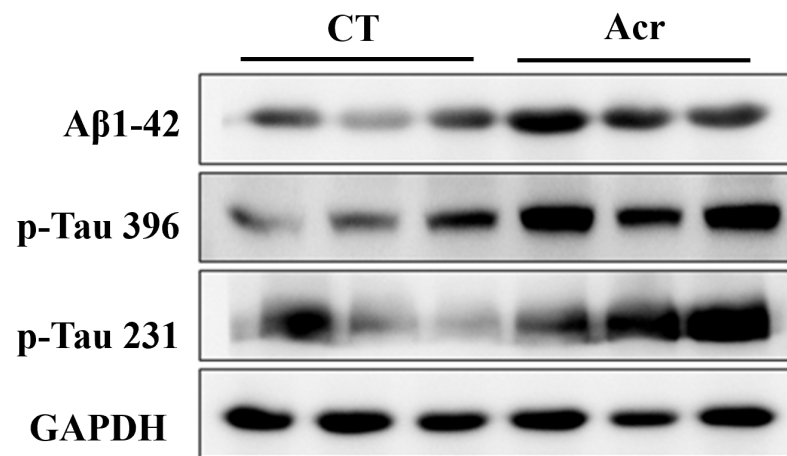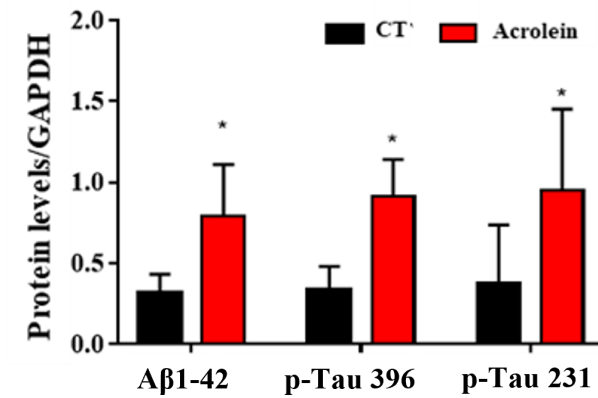

Supplement: Supplementary file 3 — Fig S2 [file ACEL-21-e13587-s001.pdf]
