# Supplementary material for: Acrolein, an endogenous aldehyde induces synaptic dysfunction in vitro and in vivo: Involvement of RhoA/ROCK2 pathway
Source: Aging Cell. 2022 Mar 22;21(4):e13587. doi: 10.1111/acel.13587 (PMC9009232; doi:10.1111/acel.13587)
Supplement: Supplementary file 5 — Supplementary Material [file ACEL-21-e13587-s004.docx]

**Supplementary figure legends**

**Figure S1. Low concentration of acrolein induces axonal rupture in primary cortical neurons.** Primary cultured rat cortical neurons were treated at DIV7 in the presence of Acrolein (5 μM) for 24 hours. Monitoring the morphological changes of neurons within 24 hours through the live cell imaging system.

**Fig S2. Acrolein increased the levels of Aβ and phosphorylated Tau proteins in hippocampus and cortex of acrolein-inducced mice.** The mice were treated with acrolein (3.0 mg/kg/d) or with distilled water for 4 weeks. After all behavior tests, the mice were scarified and their brain tissues were harvested for Western blot assay. Representative images of proteins reflecting synaptic functional expression (Aβ, p-tau 396 and p-tau 231) in the hippocampus **(A)** and cortex **(B)**  using western blot analysis. The data were expressed as mean ± SEM., n=3 *P < 0.05, **P < 0.01 vs. control group.

**Figure S3. Main DEMs induced by acrolein in primary cortical neurons by KEGG pathway analysis.** Primary cultured rat cortical neurons were treated with acrolein (5 μM) for 24 hours. (A) Axon guidance is the main pathway of DEMs induced by acrolein in primary cortical neurons in KEGG pathway analysis. (B) Glutamatergic synapse pathway of DEMs in KEGG analysis. Significant DEGs are colored yellow (up) or green (down). White or gray represent genes that are not significantly differentially expressed.
