# Supplementary material for: Acrolein, an endogenous aldehyde induces synaptic dysfunction in vitro and in vivo: Involvement of RhoA/ROCK2 pathway
Source: Aging Cell. 2022 Mar 22;21(4):e13587. doi: 10.1111/acel.13587 (PMC9009232; doi:10.1111/acel.13587)
Supplement: Supplementary file 4 — FIg S3 [file ACEL-21-e13587-s003.pdf]

A

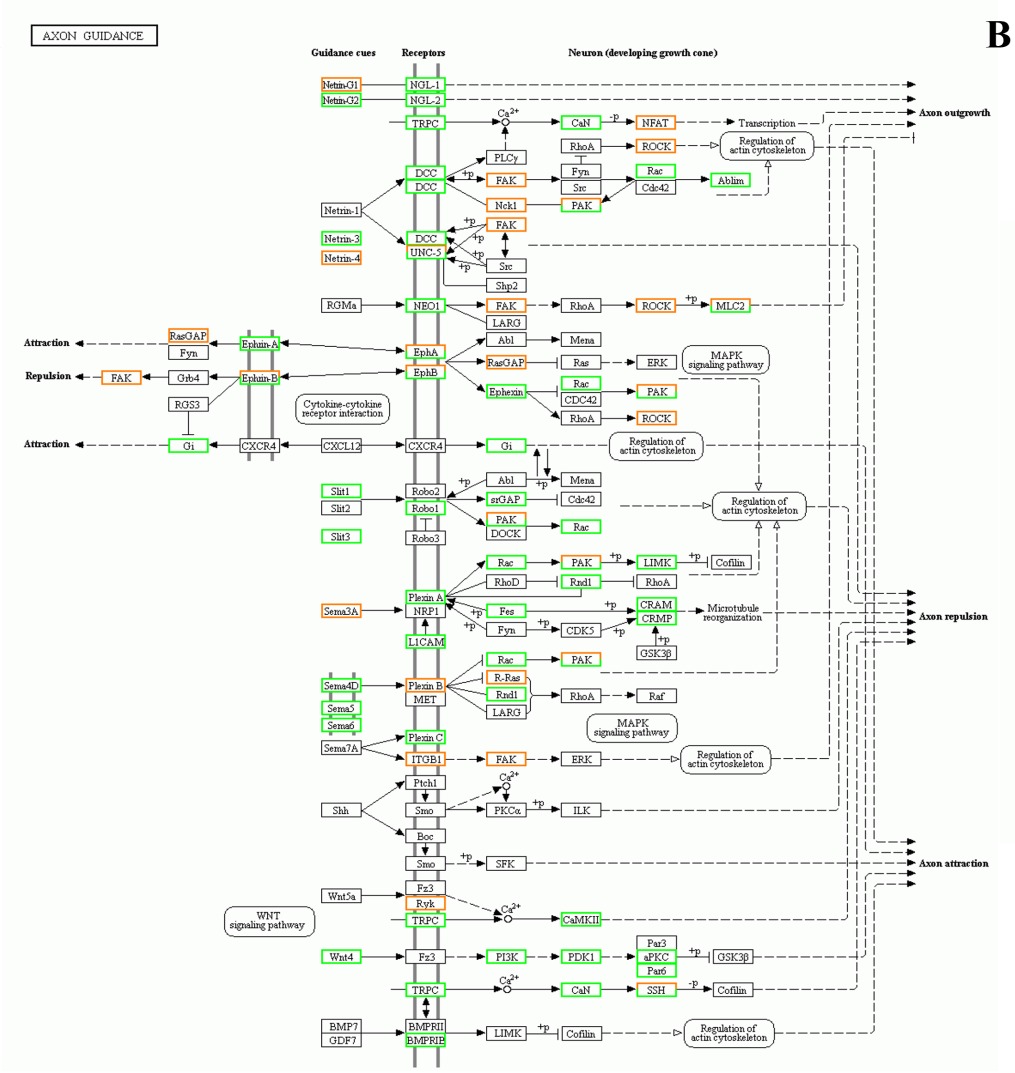
AXON GUIDANCE
Guidance cues
Receptors
Neuron (developing growth cone)
Netrin-G1
Netrin-G2
NGL-1
NGL-2
TRPC
Ca2+
CaN
NFAT
Transcription
Axon outgrowth
PLCγ
RhoA
ROCK
Regulation of actin cytoskeleton
DCC
FAK
Fyn
Src
Rac
Cdc42
Ablim
Netrin-1
Nck1
PAK
Netrin-3
Netrin-4
UNC-5
Shp2
RGMa
NEO1
MLC2
LARG
Attraction
Repulsion
RasGAP
Ephrin-A
EphA
Abl
Mena
Ras
ERK
MAPK signaling pathway
Grb4
Ephrin-B
EphB
Ephexin
CDC42
RGS3
Cytokine-cytokine receptor interaction
Gi
CXCR4
CXCL12
Slit1
Slit2
Slit3
Robo2
Robo1
Robo3
srGAP
DOCK
LIMK
Cofilin
RhoD
Rnd1
Plexin A
NRP1
L1CAM
Sema3A
Fes
CDK5
CRAM
CRMP
GSK3β
Microtubule reorganization
Axon repulsion
Sema4D
Plexin B
MET
R-Ras
Raf
Sema5
Sema6
Plexin C
Sema7A
ITGB1
Ptch1
Smo
PKCα
ILK
Shh
Boc
SFK
Axon attraction
Fz3
Wnt5a
Ryk
CaMKII
WNT signaling pathway
Wnt4
PI3K
PDK1
Par3
aPKC
Par6
SSH
BMP7
GDF7
BMPRII
BMPRIB
+p
-p

B

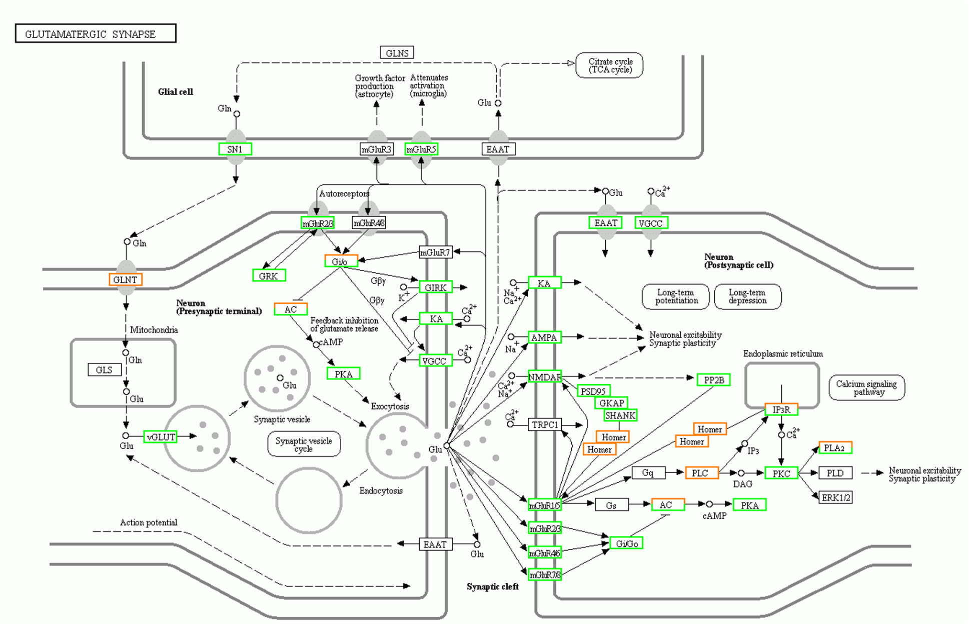
GLUTAMATERGIC SYNAPSE
GLNS
Citrate cycle (TCA cycle)
Glial cell
Growth factor production (astrocyte)
Attenuates activation (microglia)
Gln
Glu
SN1
mGluR3
mGluR5
EAAT
Autoreceptors
mGluR2/3
mGluR4/8
mGluR7
GLNT
GRK
Gi/o
Gβγ
GIRK
K+
KA
Ca2+
Na+
Neuron (Presynaptic terminal)
AC
Feedback inhibition of glutamate release
cAMP
Mitochondria
GLS
PKA
VGCC
Synaptic vesicle
Exocytosis
vGLUT
Synaptic vesicle cycle
Endocytosis
Action potential
Synaptic cleft
VGCC
Neuron (Postsynaptic cell)
Long-term potentiation
Long-term depression
AMPA
Neuronal excitability
Synaptic plasticity
NMDAR
PP2B
Endoplasmic reticulum
Calcium signaling pathway
PSD95
GKAP
SHANK
Homer
IP3R
TRPC1
Gq
PLC
IP3
DAG
PKC
PLA2
PLD
ERK1/2
mGluR1/5
Gs
mGluR4/6
mGluR7/8
Neuronal excitability Synaptic plasticity
